# Coronavirus stress and overeating: the role of anxiety and COVID-19 burnout

**DOI:** 10.1186/s40337-022-00584-z

**Published:** 2022-05-01

**Authors:** Ruining Wang, Baojuan Ye, Peiyi Wang, Chunyan Tang, Qiang Yang

**Affiliations:** 1 Center of Mental Health Education and Research, Preschool Education Research Center, School of Psychology, School of Education, Jiangxi Normal University, 99 Ziyang Avenue, Nanchang, 330022 China; 2 Department of Psychological Science, University of California, Irvine, 92617, 4201 Social and Behavioral Sciences Gateway, Irvine, CA 92697-7085 USA; 3 Health Education and Counseling Center, Jiangxi Health Vocational College, Nanchang, China

**Keywords:** Coronavirus stress, Anxiety, Overeating, COVID-burnout, College students, COVID-19

## Abstract

**Background:**

This study examined the role of anxiety and coronavirus disease 2019 (COVID-19) burnout in the relationship between coronavirus stress and overeating among Chinese college students during the COVID-19 pandemic.

**Methods:**

Chinese college students (*N* = 2926; *M*_*age*_ = 19.90, *SD* = 1.47, range = 18–25 years old; 54.34% female) completed self-reported online questionnaires regarding coronavirus stress, anxiety, COVID-19 burnout, and overeating.

**Results:**

Anxiety showed partially indirect effect on the association between coronavirus stress and overeating. COVID-19 burnout exacerbated the indirect pathway between coronavirus stress and overeating via anxiety.

**Discussion and conclusion:**

This is the first study, to our knowledge, that examines the underlying mechanisms of the coronavirus stress and overeating behavior association among Chinese college students. The results support several existing theories on stress and problematic eating behaviors and provide practical implications for prevention and intervention programs of overeating during the COVID-19 pandemic.

## Plain English summary

One’s response is not arbitrary when confronted with the coronavirus disease 2019 (COVID-19). Previous research has found that individuals with coronavirus stress still be more prone to overeat. We also know from other research that anxiety is the most salient aspect of overeating. However, no research has investigated whether the coronavirus stress of college students is significantly associated with overeating and examine the potential indirect pathway and moderating mechanisms in this association. With the aid of 2926 participants, we found that, coronavirus stress was linked to college students’ overeating. We also found that this relationship was partially explained by anxiety. In addition, the association between anxiety and overeating was stronger for those with higher COVID-19 burnout. This study is an important step in unpacking how coronavirus stress relates to overeating of Chinese college students. However, they are limited by the cross-sectional nature of the study, meaning we cannot imply causality. We recommend that further research replicate our findings in people with diagnosed feeding and eating disorders using a longitudinal design.

## Introduction

Since the outbreak of the coronavirus disease 2019 (COVID-19), research studies have documented the adverse behavioral, psychological, and physical outcomes associated with the pandemic [1–3]. One particularly concerning change in health behavior is overeating [4, 5], which is defined as the consumption of energy intake that is excessively high in comparison to one's energy expenditure [6]. While temporary overeating is typical during occasions such as feasts and celebrations [7], it becomes a health risk when sustained for an extended period. Overeating is associated with weight gain, obesity, as well as feeding and eating disorders [8]. Additionally, it is related to a series of maladaptive psychological outcomes, including internalizing and externalizing problems (e.g., depression, anxiety, self-harm, and substance use disorders) [9]. Thus, the development and maintenance of overeating poses a health concern, ultimately jeopardizing one's entire health and well-being.

Stress associated with the COVID-19 pandemic may play a substantial role in overeating. Representing a form of chronic stress, coronavirus stress could stimulate the hypothalamic–pituitary–adrenal (HPA) axis to an excessively high degree, resulting in an abnormally high glucocorticoid level in the body [10]. Meanwhile, endogenous opioid release can be induced by chronic stress because it adaptively dampens the stress response [10]; however, this process is also related to addiction and excessive food consumption [11]. A substantial amount of research has established the link between various types of stressors and overeating behavior, as well as examined the related biological mechanisms underlying the associations [12]. During the COVID-19 pandemic, a small number of studies have reported a direct positive association between coronavirus stress and problematic eating patterns [13, 14].

While the direct link between coronavirus stress and problematic eating patterns has been established, the exact psychological mechanism by which the link occurs remains unclear. Anxiety could be one of these mechanisms. Anxiety, representing a longer-term psychological process [15], is defined as an emotional reaction to stress that involves fear, apprehension, a sense of danger, and persistent thoughts and worries [15]. When individuals are unable to manage stressful events, their risk of developing anxiety is aggravated [16]. Increased mental health issues, including anxiety symptoms associated with the COVID-19 pandemic, have been recorded [17], and stress, in particular, has been particularly constructed as a predictor of anxiety [18]. Anxiety, in turn, is associated with adverse health outcomes [19] and overeating, in particular [20, 21]. In terms of problematic eating, the evidence consistently demonstrates a link between anxiety and overeating [22, 23], probably because of the lack of control, negative arousal, and body-image related factors. Furthermore, though a range of negative affective states (e.g., depression and anger) are theoretically associated with overeating, the link between overeating and anxiety was remarkably more salient than that and other negative affective states [24]. Therefore, with the probable links between coronavirus stress and anxiety, and between anxiety and overeating, anxiety could be one psychological mechanism underlying the direct association between coronavirus stress and overeating.

Burnout, a distinct psychological construct from anxiety [26], during the COVID-19 pandemic might further exacerbate the link between anxiety and overeating. Burnout refers to a psychological condition generated by an extended response to interpersonal pressures [25] and is manifested by emotional exhaustion, feelings of cynicism, and diminished personal success [26]. The risk enhancing model indicates that while the role of a single risk is relatively limited in predicting health outcomes, when the risk accumulates, one risk factor may exacerbate the role of another, resulting in severe mental health consequences [27, 28]. The association between general burnout and overeating has been reported [29]. During the COVID-19 pandemic, studies have documented increased overeating [30]. Thus, COVID-19 related burnout might be a relevant construct in the coronavirus stress and overeating association, and might function to enhance the indirect effect of anxiety in the link between coronavirus stress and overeating.

**Fig. 1:**
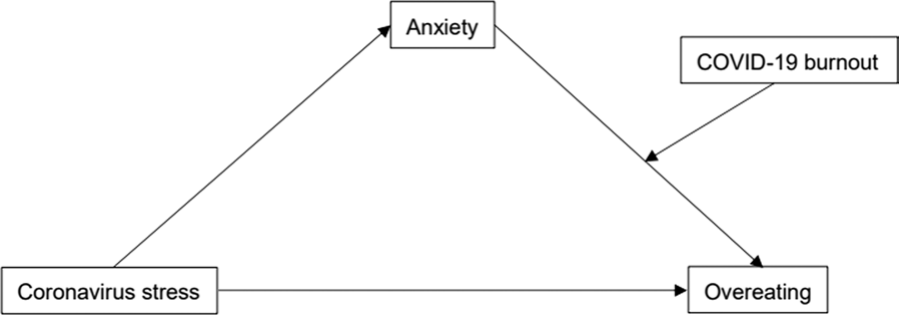
The proposed moderated model

College students, in general, represent a vulnerable population to the COVID-19 pandemic [18, 31], and they suffer from unhealthy eating habits [32, 33]. However, because problematic eating is studied primarily among college students in the Western culture and cultural context is critical when examining psychological issues [34], it is critical to further the understanding of eating behavior in other cultural contexts, such as in the Chinese college student population. Therefore, the purposes of this study were to examine: (1) the direct association between coronavirus stress and overeating among Chinese college students; (2) the indirect effect of anxiety underlying this link; and (3) the moderating role of COVID-19 burnout in the indirect pathway. Coronavirus stress and overeating were expected to be positively correlated (hypothesis 1) and partially linked via anxiety (hypothesis 2). Furthermore, the positive link between anxiety and overeating was predicted to be strengthened by COVID-19 burnout. The indirect effect of anxiety would therefore be more pronounced with higher COVID-19 burnout (hypothesis 3). Figure 1 depicts the proposed study model.

## Method

### Participants

This study surveyed 2,926 Chinese college students (*M*_age_ = 19.90, *SD* = 1.47, range = 18–25 years old; 54.34% female). The majority of the participants (37.80%) were first-year students, followed by 28.02% second-year, 17.40% third-year, and 16.78% fourth-year students. None of the participants lived on campus because of the COVID-19 policy.

### Procedure

Participants were recruited from more than 30 universities in 26 regions, such as Jiangxi, Jilin, and Shandong provinces of China. Universities participating in the study included those classified as Double First Class and those classified as ordinary. Data was collected from February 9 to February 13, 2020. The sampling error was calculated using the website (https://www.sphanalytics.com/sample-error-calculator/). This sample error of this research was 1.5%, which is considered within the acceptable limit [35].

Convenience sampling strategies were used. To collect data, we first created an online survey using Survey Star (i.e., an online survey distribution website; Changsha Ranxing Science and Technology, Shanghai, China), and then generated a QR code for easy distribution. Subsequently, the QR code was sent to WeChat and QQ groups (i.e., online social media platforms) to which enrolled university students had exclusive access. Students were then able to read the study information sheet and indicate informed consent prior to answering the questionnaires at their preferred location and time.

This study was approved by the Research Ethics Committee of the first author’s institution. No identifiable information was recorded. Students were instructed that participation in this study was fully voluntary and that they could exit the survey at any time. Participants received no compensation for their participation.

### Measures

#### Coronavirus stress

Coronavirus stress was assessed by the Coronavirus Stress Measure (CSM) [36]. The scale has 5 items (e.g., “how often have you felt that you were unable to control the important things in your life due to the COVID-19 pandemic?”). Participants rated each item on a five-point scale ranging from 0 (*never*) to 4 (*always*), with higher scores showing higher levels of coronavirus stress. In this study, the internal consistency coefficient was 0.93. Confirmatory factor analysis (CFA) in this study suggested that the one-factor model fit the data well: RMSEA = 0.05, CFI = 0.99, TLI = 0.99, SRMR = 0.01, indicating good scale validity [37, 38].

#### Anxiety

The Chinese-version of the anxiety subscale of the Depression Anxiety Stress Scale (DASS-21) [39], adapted from Lovibond and Lovibond [40], was used. This scale consists of 7 items (e.g., “I felt I was close to panic.”). Participants rated each item on a four-point scale ranging from 0 (*very inconsistent*) to 3 (*very consistent*), with higher scores showing higher levels of anxiety. The scale has good reliability, validity, and applicability to the Chinese population [41, 42]. In this study, the internal consistency coefficient was 0.89. Confirmatory factor analysis (CFA) in this study suggested that the one-factor model fit the data well: RMSEA = 0.06, CFI = 0.99, TLI = 0.98, SRMR = 0.01.

#### COVID-19 burnout

The COVID-19 burnout was assessed by the COVID-19 burnout scale [42]. This scale has 10 items (e.g., “When you think about COVID-19 overall, how often do you feel disappointed with people?”). Participants rated each item on a five-point scale ranging from 1 (*never*) to 5 (*always*), with higher scores showing higher levels of COVID-19 burnout. In this study, the internal consistency coefficient was 0.93. Confirmatory factor analysis (CFA) in this study suggested that the one-factor model fit the data well: RMSEA = 0.07, CFI = 0.99, TLI = 0.97, SRMR = 0.01, which indicated that the validity of this scale was good [37, 38].

#### Overeating

The Chinese version of overeating [43] was measured by the loss of control eating subscale of the Three-Factor Eating Scale [44]. This scale comprises 9 items (e.g., “when I smell a delicious food, I find it very difficult to keep from eating, even if I have just finished a meal.”), and all questions were asked specifically to the period after the COVID-19 pandemic outbreak. Participants rated each item on a four-point scale ranging from 1 (*very inconsistent*) to 4 (*very consistent*), with higher scores showing higher levels of overeating. The scale has good reliability, validity, and applicability to the Chinese population [45, 46]. In this study, the internal consistency coefficient was 0.94. Confirmatory factor analysis (CFA) in this study suggested that the one-factor model fit the data well: RMSEA = 0.04, CFI = 0.99, TLI = 0.99, SRMR = 0.01, which indicated that the validity of this scale was good [37, 38].

### Data analyses strategy

First, the descriptive statistics and bivariate Pearson correlations were calculated among the study variables using SPSS (version 23). Second, the hypothesized indirect effect of anxiety was examined using Model 4 of the PROCESS macro [47] in SPSS. Third, the hypothesized moderating effect of COVID-19 burnout on the indirect link between coronavirus stress and overeating via anxiety was tested using Model 14 of the PROCESS macro in SPSS. All study continuous variables were standardized prior to analyses. The indirect pathway and moderation models were estimated with 5000 bootstrap resamples to produce bias-corrected confidence intervals [47]. Because only completed surveys were recorded, there is no missing data in the current dataset.

## Results

### Preliminary analyses

The means and Pearson-correlations among the study variables are presented in Table 1. Coronavirus stress was related to greater anxiety (*r* = 0.54), overeating (*r* = 0.34), and COVID-19 burnout (*r* = 0.48; *all ps* < 0.001), supporting hypothesis 1. Anxiety was associated with greater COVID-19 burnout (*r* = 0.64, *p* < 0.001) and overeating (*r* = 0.48, *p* < 0.001) and. Lastly, COVID-19 burnout was related to higher overeating (*r* = 0.44, *p* < 0.001).

**Table 1:** Descriptive statistics and correlations among study variables (N = 2926)

Variable	*M*	*SD*	1	2	3
1. Coronavirus stress	2.27	0.81	–		
2. Anxiety	0.77	0.54	0.54***	–	
3. COVID-19 burnout	2.44	0.66	0.48***	0.64***	–
4. Overeating	2.32	0.54	0.34***	0.48***	0.44***

^***^p < 0.001

### Anxiety as an indirect pathway

Coronavirus stress was related to higher anxiety (*β* = 0.54, *p* < 0.001) and anxiety was further related to greater overeating (*β* = 0.42, *p* < 0.001). The residual direct effect of coronavirus stress on overeating remained positive (*β* = 0.11, *p* < 0.001), but the strength of this association was reduced compared to the total effect (*β* = 0.34, *p* < 0.001). These results showed that anxiety showed partially indirect effect on the association between coronavirus stress and overeating (indirect effect = 0.23, *SE* = 0.015, 95% CI [0.20, 0.26]), and the indirect effect accounted for 66.84% of the total effect of coronavirus stress on overeating. Therefore, hypothesis 2 was supported (Table 2).

**Table 2:** The indirect effect of anxiety on the association between coronavirus stress (CS) and overeating

Predictor	Anxiety		Overeating	
	*β*	*t*	*β*	*t*
CS	0.54	34.74***	0.11	5.83***
Anxiety			0.42	21.75***
*R*^2^	0.29		0.24	
*F*	1206.84***		455.05***	

*N* = 2926. Each column is a regression model that forecasts the criterion at the top of the column

^***^p < 0.001

### The moderation effect of COVID-19 burnout in the indirect association between coronavirus stress and overeating via anxiety

The direct effect of coronavirus stress on overeating was significant (*β* = 0.06, *p* < 0.001). With anxiety and the interaction term (anxiety × COVID-19 burnout) included in the model, the direct effect of coronavirus stress on overeating remained significant with a smaller effect size. Furthermore, anxiety was, again, significant related to coronavirus stress (*β* = 0.54, *p* < 0.001) and overeating (*β* = 0.27, *p* < 0.001. The interaction term of anxiety and COVID-19 burnout showed a significant positive association with overeating (*β* = 0.06, *p* < 0.001), suggesting the indirect effect of anxiety on overeating was moderated by COVID-19 burnout (Table 3).

**Table 3:** Testing the moderation effect of COVID-19 burnout in the indirect association

Predictor	Anxiety		Overeating	
	*β*	*t*	*β*	*t*
CS	0.54	34.74***	0.06	3.37***
Anxiety			0.27	11.65***
Anxiety × CB			0.06	5.21***
*R*^2^	0.29		0.27	
*F*	1206.84***		270.43***	

*N* = 2926. Each column is a regression model that forecasts the criterion at the top of the column

*CS* coronavirus stress, *CB* COVID-19 Burnout

^**^p < 0.01, ***p < 0.001

**Fig. 2:**
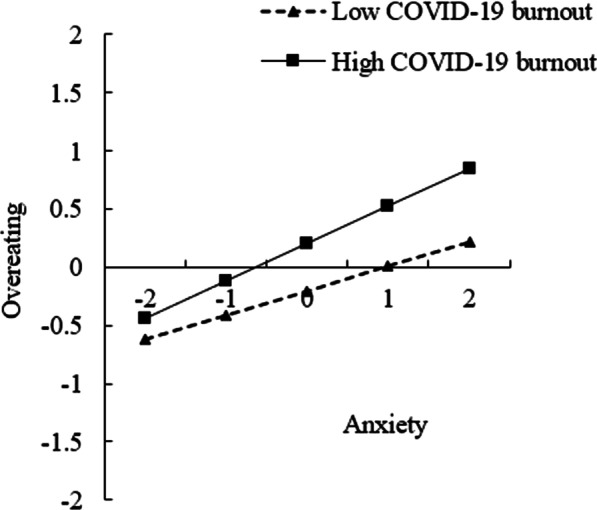
Interaction figure for indirect paths

We subsequently plotted predicted overeating against anxiety, separately for low and high levels of COVID-19 burnout (one SD below the mean and one SD above the mean, respectively; Fig. 2). The simple slope test demonstrated that for college students with low levels of COVID-19 burnout, anxiety was positively associated with overeating, *β* = 0.21, *p* < 0.001. In contrast, for college students with high COVID-19 burnout, anxiety yielded a stronger positive association with overeating, *β* = 0.33, *p* < 0.001. Thus, COVID-19 burnout strengthened the association between anxiety and overeating.

The bias-corrected percentile bootstrap analysis further indicated that the indirect effect of coronavirus stress on overeating through anxiety was moderated by COVID-19 burnout. Particularly, for college students low in COVID-19 burnout, the indirect effect of coronavirus stress on overeating via anxiety was significant, *β* = 0.21, *SE* = 0.03, 95% CI_boot_ [0.16, 0.27]. Comparatively, for college students with high COVID-19 burnout, the indirect effect was stronger, *β* = 0.33, *SE* = 0.02, 95% CI_boot_ [0.28, 0.37]. Thus, supporting hypothesis 3, results indicated that the indirect effect of anxiety was exacerbated by COVID-19 burnout.

## Discussion

The current study suggests that the experience of coronavirus stress is associated with increased overeating among Chinese college students. Anxiety is one of the mechanisms underlying this relationship, while COVID-19 burnout moderates the indirect effect of anxiety. COVID-19 burnout, in particular, strengthens the link between anxiety and overeating. While several empirical studies have reported the association between coronavirus stress and overeating [48, 49], the current study is the first, to our knowledge, to examine the underlying psychological mechanisms of this link among Chinese college students.

### Anxiety as an indirect pathway

Anxiety represents a partial indirect pathway linking coronavirus stress and overeating. The positive correlation between coronavirus stress and anxiety is consistent with empirical evidence that both perceived stress and anxiety have increased significantly during the pandemic [50, 51], as well as the biological mechanisms underlying this association [52]. It further lends credence to the proposition that stress related to the COVID-19 pandemic might be a strong predictor of diagnosable psychiatric disorders, including anxiety [53]. Due to the lack of social connections and physical activities during the COVID-19 pandemic, individuals who experience stress may have trouble managing their emotions, resulting in increased internationalizing symptoms such as anxiety [54].

Importantly, anxiety from coronavirus stress was connected with overeating in the current study. This is consistent with the escape model [55], which postulates that overeating is a coping mechanism for individuals seeking to escape from negative self-perceptions by narrowing attention to the unpleasant situation and thus disinhibiting food intake. While overeating may provide momentary relief or pleasure [56–58], it can also result in a number of negative mental and physical health consequences, including weight gain, obesity, and eating disorders. Our finding thus suggests that proper stress coping strategies and anxiety management skills might benefit Chinese students who develop overeating issues during the pandemic.

It is also worth noting that the direct positive link between coronavirus stress and overeating persisted, even after anxiety was considered in the model. This remaining link implies that, in addition to the psychological disorder symptoms associated with coronavirus stress, coronavirus stress itself might be a distinct and significant risk factor for overeating. This outcome is likely given the endocrine and other pertinent biological mechanisms underpinning the relationship between chronic stress and binge eating disorder, a condition characterized by excessive eating [59]. Moreover, there might be other factors within this association. For instance, weight concerns, emotional eating tendencies, and appearance overevaluation are all related to overeating and stress [60, 61] and thus could be relevant mechanisms for the current study topic.

### COVID-19 burnout as a moderator

COVID-19 burnout exacerbated the link between anxiety and overeating, hence intensifying the indirect pathway of anxiety in the coronavirus stress and overeating association. Increased levels of burnout, characterized by greater emotional tiredness and depersonalization, as well as lower personal accomplishment, have been documented during this COVID-19 pandemic [62]. Adding to prior research revealing that burnout and overeating can coexist [63, 64], our finding further alters that the experience of COVID-19 burnout might be associated with poorer dieting habits when Chinese college students experience high anxiety.

### Strengths

This study extends prior research on coronavirus stress and overeating by examining the underlying psychological mechanisms during the COVID-19 pandemic in the Chinese college student population. The findings concur with a number of theories and empirical studies on stress, anxiety, and poor eating patterns, particularly in light of the COVID-19 pandemic. More importantly, our findings have practical implications. Overeating prevention and treatment programs may find it effective to focus on decreasing coronavirus stress, anxiety, and COVID-19 burnout. For example, mindfulness-based stress reduction courses could be evaluated because they have been shown to reduce stress and burnout through improved attention management and tolerance for negative emotions [65, 66]. Exercise intervention may be beneficial in lowering college students' anxiety levels [67, 68], hence reducing overeating. Additionally, while screening Chinese college students' susceptibility to stress, anxiety, and burnout during the pandemic, health professionals may find it advantageous to analyze their dietary habits concurrently to initiate appropriate early intervention for problematic eating behaviors.

### Limitations and future directions

The following limitations should be considered when interpreting the findings of this study. First, this study utilized a cross-sectional design, which prevents causal inferences. While the current moderated indirect pathway model is suggestive [69], longitudinal investigations are necessary to compare the current findings [69]. Furthermore, individuals self-reported their emotions, which may not accurately reflect their psychological states [70]. Other research methodologies, such as interviews, direct observation, or big data analysis, may serve to further elucidate the relationships between stress, anxiety, and overeating. Additionally, personality traits, such as neuroticism, which underpin an individual's susceptibility to unpleasant feelings, may be relevant to the current study topic [71, 72], and should be examined in future research. Lastly, potential confounders, such as eating disorder status [73], food insecurity [74], and living status [75], should be examined in the current study topic.

## Conclusion

This study is critical in elucidating the relationship between coronavirus stress and overeating among Chinese college students. Because anxiety represents an indirect pathway in this direct association and COVID-19 burnout exacerbates the link between anxiety and overeating, our study emphasizes the critical nature of addressing anxiety and COVID-19 burnout in overeating prevention and intervention programs. Further, coronavirus stress itself represents a direct risk factor for overeating, and, thus, should be considered in these programs. As the COVID-19 pandemic continues, it is critical to monitor college students' psychological well-being because its link to recurrent overeating may pose long-term health problems.

## Data Availability

This study data is available to researchers.

## Abbreviations

CS: Coronavirus stress
CB: COVID-19 burnout

## References

[1] Dubey S, Biswas P, Ghosh R, Chatterjeed S, Dubeye MJ, Chatterjeeet S (2020). Psychosocial impact of COVID-19. Diabetes Metab Syndr.

[2] Pedrosa AL, Bitencourt L, Fróes ACF, Cazumbá MLB, Campos RGB, de Brito SBCS (2020). Emotional, behavioral, and psychological impact of the COVID-19 pandemic. Front Psychol.

[3] Stockwell S, Trott M, Tully M, Shin J, Barnett Y, Butler L (2021). Changes in physical activity and sedentary behaviours from before to during the COVID-19 pandemic lockdown: a systematic review. BMJ Open Sport Exerc Med.

[4] Scarmozzino F, Visioli F (2020). COVID-19 and the subsequent lockdown modified dietary habits of almost half the population in an Italian sample. Foods.

[5] Sidor A, Rzymski P (2020). Dietary choices and habits during COVID-19 lockdown: experience from Poland. Nutrients.

[6] Prentice AM (2001). Overeating: the health risks. Obes Res.

[7] Prentice AM, Diaz E, Goldberg GR, Jebb SA, Coward WA, Whitehead RG, Anderson GH, Kennedy SH (1992). Famine and refeeding: adaptations in energy metabolism. The biology of feast and famine: relevance to eat disord.

[8] Goldschmidt AB, Loth KA, MacLehose RF, Pisetsky EM, Berge JM, Neumark-Sztainer D (2015). Overeating with and without loss of control: associations with weight status, weight-related characteristics, and psychosocial health. Int J Eat Disord.

[9] Muratori F, Viglione V, Maestro S, Picchi L (2004). Internalizing and externalizing conditions in adolescent anorexia. Psychopathology.

[10] Adam TC, Epel ES (2007). Stress, eating and the reward system. Physiol Behav.

[11] Karkhanis A, Holleran KM, Jones SR (2017). Dynorphin/kappa opioid receptor signaling in preclinical models of alcohol, drug, and food addiction. Int Rev Neurobiol.

[12] Anversa RG, Muthmainah M, Sketriene D, Gogos A, Sumithran P, Brown RM (2021). A review of sex differences in the mechanisms and drivers of overeating. Front Neuroendocrinol.

[13] Khubchandani J, Kandiah J, Saiki D (2020). The COVID-19 pandemic, stress, and eating practices in the United States. Eur J Investig Health Psychol Educ.

[14] Smith KR, Jansen E, Thapaliya G, Aghababian AH, Chen L, Sadler JR, Carnell S (2021). The influence of COVID-19-related stress on food motivation. Appetite.

[15] Zysberg L (2018). Emotional intelligence, anxiety, and emotional eating: A deeper insight into a recently reported association?. Eat Behav.

[16] Pappa S, Ntella V, Giannakas T, Giannakoulis VG, Papoutsi E, Katsaounou P (2020). Prevalence of depression, anxiety, and insomnia among healthcare workers during the COVID-19 pandemic: a systematic review and meta-analysis. Brain Behav Immun.

[17] Salari N, Hosseinian-Far A, Jalali R, Vaisi-Raygani A, Rasoulpoor S, Mohammadi M (2020). Prevalence of stress, anxiety, depression among the general population during the COVID-19 pandemic: a systematic review and meta-analysis. Glob Health.

[18] Varma P, Junge M, Meaklim H, Jackson ML (2021). Younger people are more vulnerable to stress, anxiety and depression during COVID-19 pandemic: a global cross-sectional survey. Prog Neuropsychopharmacol Biol Psychiatry.

[19] Hoffman DL, Dukes EM, Wittchen HU (2008). Human and economic burden of generalized anxiety disorder. Depress Anxiety.

[20] Rosenbaum DL, White KS (2016). Does cognitive avoidance mediate the relation of anxiety and binge eating?. Eat Weight Disord.

[21] Rosenbaum DL, White KS (2015). The relation of anxiety, depression, and stress to binge eating behavior. J Health Psychol.

[22] Graff C, Pearcey SM, Zhan G (2021). General anxiety and overeating in undergraduate students. Kennesaw J Undergrad Res.

[23] Slochower J, Kaplan SP (1980). Anxiety, perceived control, and eating in obese and normal weight persons. Appetite.

[24] Macht M, Simons G (2000). Emotions and eating in everyday life. Appetite.

[25] Maslach C, Leiter MP (2016). Understanding the burnout experience: recent research and its implications for psychiatry. WPA.

[26] Koutsimani P, Montgomery A, Georganta K (2019). The relationship between burnout, depression, and anxiety: a systematic review and meta-analysis. Front Psychol.

[27] Bao ZY, Li DP, Zhang W, Wang YH, Sun WQ, Zhao LY (2014). Cumulative ecological risk and adolescents’ academic and social competence: the compensatory and moderating effects of sense of responsibility to parents. Psychol Dev Educ.

[28] Li DP (2012). Multiple ecological risk factors and adolescents’ social adaptation: how risks should be modeled and what are their mechanisms.

[29] Nevanperä NJ, Hopsu L, Kuosma E, Ukkola O, Uitti J, Laitinen JH (2012). Occupational burnout, eating behavior, and weight among working women. Am J Clin Nutr.

[30] Gonzalez-Ramirez J, Mulqueen K, Zealand R, Silverstein S, Mulqueen C, BuShell S (2021). Emergency online learning: college students' perceptions during the COVID-19 pandemic. Coll Stud J.

[31] Li Y, Zhao J, Ma Z, McReynolds LS, Lin D, Chen Z (2021). Mental health among college students during the COVID-19 pandemic in China: a 2-wave longitudinal survey. J Affect Disord.

[32] Fitzsimmons-Craft EE, Karam AM, Monterubio GE, Taylor CB, Wilfley DE (2019). Screening for eating disorders on college campuses: a review of the recent literature. Curr Psychiatry Rep.

[33] Stok FM, Renner B, Clarys P, Lien N, Lakerveld J, Deliens T (2018). Understanding eating behavior during the transition from adolescence to young adulthood: a literature review and perspective on future research directions. Nutrients.

[34] Doris E, Shekriladze I, Javakhishvili N, Jones R, Treasure J, Tchanturia K (2015). Is cultural change associated with eating disorders? A systematic review of the literature. Eat Weight Disord.

[35] Gleaves DH, Pearson CA, Ambwani S, Morey LC (2014). Measuring eating disorder attitudes and behaviors: a reliability generalization study. J Eat Disord.

[36] Arslan G, Yıldırım M, Tanhan A, Bulus M, Allen K (2020). Coronavirus stress, optimism–pessimism, psychological inflexibility, and psychological health: psychometric properties of the coronavirus stress measure. Int J Ment Health Addict.

[37] Brown TA (2006). Confirmatory factor analysis for applied research.

[38] Mulaik SA (2009). Linear causal modeling with structural equations.

[39] Gong X, Xie XY, Xu R, Luo JY (2010). Psychometric properties of the Chinese versions of DASS-21 in Chinese college students. Chin J Clin Psychol.

[40] Lovibond SH, Lovibond PF (1996). Manual for the depression anxiety stress scales.

[41] Han T, Ma WD, Gong H, Hu YC, Zhang Y, Zhang CY (2021). Investigation and analysis of negative emotion among university students during home quarantine of COVID-19. J Xi'an Jiaotong Univ.

[42] Murat Y, Fatma S (2020). COVID-19 burnout, Coronavirus stress and resilience: Initial psychometric properties of COVID-19 burnout scale. Death Stud.

[43] Luo YJ. Effects of early life environmental unpredictability on overeating. Southwest Univ.; 2020.

[44] Stunkard AJ, Messick S (1985). The three-factor eating questionnaire to measure dietary restraint, disinhibition and hunger. J Psychosom Res.

[45] Chen XM, Luo YJ, Chen H (2020). Friendship quality and adolescents’ intuitive eating: a serial mediation model and the gender difference. Acta Psychol Sin.

[46] Luo YJ, Niu GF, Chen H (2020). Early life environmental unpredictability and overeating: based on life history theory. Acta Psychol Sin.

[47] Hayes AF (2017). Introduction to mediation, moderation, and conditional process analysis: a regression-based approach.

[48] Modrzejewska A, Czepczor-Bernat K, Modrzejewska J, Matusik P (2021). Eating motives and other factors predicting emotional overeating during COVID-19 in a sample of Polish adults. Nutrients.

[49] Sadler JR, Thapaliya G, Jansen E, Aghababian AH, Smith KR, Carnell S (2021). COVID-19 stress and food intake: protective and risk factors for stress-related palatable food intake in US adults. Nutrients.

[50] Bareeqa SB, Ahmed SI, Samar SS, Yasin W, Zehra S, Monese GM (2021). Prevalence of depression, anxiety and stress in China during COVID-19 pandemic: a systematic review with meta-analysis. Int J Psychiatry Med.

[51] Husky MM, Kovess-Masfety V, Swendsen JD (2020). Stress and anxiety among university students in France during COVID-19 mandatory confinement. Compr Psychiatry.

[52] Slavich GM (2020). Social safety theory: a biologically based evolutionary perspective on life stress, health, and behavior. Annu Rev Clin Psychol.

[53] Gruber J, Prinstein MJ, Clark LA, Rottenberg J, Abramowitz JS, Albano AM (2020). Mental health and clinical psychological science in the time of COVID-19: challenges, opportunities, and a call to action. Am Psychol.

[54] Brehl AK, Kohn N, Schene AH, Fernández G (2020). A mechanistic model for individualized treatment of anxiety disorders based on predictive neural biomarkers. Psychol Med.

[55] Heatherton TF, Baumeister FF (1991). Binge eating as escape from self-awareness. Psychol Bull.

[56] Goossens L, Braet C, Van Vlierberghe L, Mels S (2009). Loss of control over eating in overweight youngsters: the role of anxiety, depression and emotional eating. Eur Eat Disord Rev.

[57] Sulkowski ML, Dempsey J, Dempsey AG (2011). Effects of stress and coping on binge eating in female college students. Eat Behav.

[58] Leehr EJ, Krohmer K, Schag K, Dresler T, Zipfel S, Giel KE (2015). Emotion regulation model in binge eating disorder and obesity—a systematic review. Neurosci Biobehav Rev.

[59] Razzoli M, Pearson C, Crow S, Bartolomucci A (2017). Stress, overeating, and obesity: insights from human studies and preclinical models. Neurosci Biobehav Rev.

[60] Schulte SJ (2016). Predictors of binge eating in male and female youths in the United Arab Emirates. Appetite.

[61] Stice E, Presnell K, Spangler D (2002). Risk factors for binge eating onset in adolescent girls: a 2-year prospective investigation. Health Psychol.

[62] Pappa S, Athanasiou N, Sakkas N, Patrinos S, Sakka E, Barmparessou Z, Tsikrika S, Adraktas A, Pataka A, Migdalis I, Gida S, Katsaounou P (2021). From recession to depression? Prevalence and correlates of depression, anxiety, traumatic stress and burnout in healthcare workers during the COVID-19 pandemic in Greece: a multi-center, cross-sectional study. Int J Environ Res Public Health.

[63] Pena Gralle APB, Barbosa Moreno A, Lopes Juvanhol L, Mendes da Fonseca MJ, Prates Melo EC, Antunes Nunes MA (2017). Job strain and binge eating among Brazilian workers participating in the ELSA-Brasil study: Does BMI matter?. J Occup Health.

[64] Medisauskaite A, Kamau C (2019). Does occupational distress raise the risk of alcohol use, binge-eating, ill health and sleep problems among medical doctors? A UK cross-sectional study. BMJ Open.

[65] Wang SX, Zheng RM, Sun MY, Zhao DF, Yang M (2021). Evaluation on effect of mindfulness-based stress reduction courses on job burnout of medical staff. Med Soc.

[66] Wu YX, Zhou T, Fang WL, Huang Z (2021). Effect of short training course of mindfulness-based stress self-help (MBSS) on job burnout in psychiatric nurses. Chin Ment Health J.

[67] DeBoer LB, Powers MB, Utschig AC, Otto MW, Smits JAJ (2012). Exploring exercise as an avenue for the treatment of anxiety disorders. Expert Rev Neurother.

[68] He C (2022). Meta-analysis of the effects of exercise intervention on college students' anxiety. Sichuan Sports Sci.

[69] Maxwell SE, Cole DA, Mitchell MA (2011). Bias in cross-sectional analyses of longitudinal mediation: partial and complete mediation under an autoregressive model. Multivar Behav Res.

[70] Kihlstrom JF, Eich E, Sandbrand D, Tobias BA, Stone AA, Turkkan JS, Bachrach CA, Jobe JB, Kurtzman HS, Cain VS (1999). Emotion and memory: Implications for self-report. The science of self-report.

[71] Khosravi M (2020). The challenges ahead for patients with feeding and eating disorders during the COVID-19 pandemic. J Eat Disord.

[72] Khosravi M (2020). Neuroticism as a marker of vulnerability to COVID-19 infection. Psychiatry Investig.

[73] Goldschmidt AB, Engel SG, Wonderlich SA, Crosby RD, Peterson CB, Le Grange D (2012). Momentary affect surrounding loss of control and overeating in obese adults with and without binge eating disorder. Obesity.

[74] Stinson EJ, Votruba SB, Venti C, Perez M, Krakoff J, Gluck ME (2018). Food insecurity is associated with maladaptive eating behaviors and objectively measured overeating. Obesity.

[75] Owen AJ, Tran T, Hammarberg K, Kirkman M, Fisher J (2021). COVID-19 restrictions impact research group. Poor appetite and overeating reported by adults in Australia during the coronavirus-19 disease pandemic: A population-based study. Public Health Nutr.

